# Supplementary material for: Differential impact of admission type and clinical complexity on diabetes hospitalization costs among African American and hispanic patients in Southeastern Virginia
Source: PLoS One. 2026 Feb 11;21(2):e0342483. doi: 10.1371/journal.pone.0342483 (PMC12893543; doi:10.1371/journal.pone.0342483)
Supplement: S1 Table — ***p < 0.001, **p < 0.01, *p < 0.05 Note: Coefficients represent percentage change in charges per unit increase. (DOCX) [file pone.0342483.s001.docx]

**Supplement**:

**S1 Table. Continuous Predictors of Healthcare Costs: Effect Size per Unit Increase**

|  | **──────── % Increase in Costs (95% CI) ────────** | | | |
| --- | --- | --- | --- | --- |
| ***Predictor*** | ***DCC*** | ***DMCC*** | ***DWO*** | |
| Length of Stay (per day) | 17.2***(16.5-17.9) | 15.1*** (14.4-15.8) | 17.4*** (15.9-8.9) | |
| Age (per year) | 0.3***(0.2-0.4) | 0.3* (0.1-0.5) | -0.1 (-0.3-0.1) | |
| Comorbidities (per condition) | 7.5*** (6.7-8.3) | 6.4*** (5.5-7.3) | 2.8 (0.1-5.5) | |
| ***p<0.001, **p<0.01, *p<0.05 Note: Coefficients represent percentage change in charges per unit increase | | | |  |
